# Impacts of Participation in Community-Based Physical Activity Programs on Cognitive Functions of Children and Youth with Neurodevelopmental Disabilities: A Scoping Review

**DOI:** 10.3390/brainsci11020195

**Published:** 2021-02-05

**Authors:** Mojgan Gitimoghaddam, Leigh M. Vanderloo, Rebecca Hung, Andrea Ryce, William McKellin, Anton Miller, Jean-Paul Collet

**Affiliations:** 1 Department of Pediatrics, University of British Columbia, Vancouver, BC V6H 0B3, Canada; mgitimoghaddam@bcchr.ca (M.G.); amiller@cw.bc.ca (A.M.); 2 BC Children’s Hospital Research Institute, Vancouver, BC V5Z 4H4, Canada; 3 Department of Knowledge Translation, ParticipACTION, Toronto, ON M5S 1M2, Canada; lvande32@uwo.ca; 4 Child Health Evaluative Sciences, The Hospital for Sick Children, Toronto, ON M5G 0A4, Canada; 5 Faculty of Science, University of British Columbia, Vancouver, BC V6T 1Z4, Canada; rebecca.hung@ubc.ca; 6 Sunny Hill Health Centre for Children, Vancouver, BC V6H 3N1, Canada; Andrea.Ryce@cw.bc.ca; 7 Department of Anthropology, University of British Columbia, Vancouver, BC V6T 1Z1, Canada; w.mckellin@ubc.ca

**Keywords:** physical activity, cognition, neurodevelopmental disorders, children and youth

## Abstract

This review paper aimed to undertake an extensive exploration of the extent, range, and nature of research activities regarding the effect and emerging evidence in the field of physical activity interventions on cognitive development among children and youth (0–17.99 years) with neurodevelopmental disorders (NDD), and to help identify key gaps in research and determine precise research questions for future investigations. To carry out this scoping review, five electronic databases were searched. A total of 12,097 articles were retrieved via search efforts with an additional 93 articles identified from the identified review papers. Sixty articles were eligible for inclusion. The results of this scoping review revealed many positive key cognitive outcomes related to physical activity including, but not limited to: focus, attention, self-control, cognitive process, and alertness. No studies reported a negative association between physical activity and cognitive outcomes. Based on the findings from this scoping review, physical activity appears to have a favorable impact on the cognitive outcomes of children and youth with NDD.

## 1. Introduction

The prevalence of neurodevelopmental disabilities (NDD) among children continues to grow. In the United States and Canada, approximately 10–17% of the pediatric population (4–14 years) is diagnosed with NDD [1] including attention-deficit/hyperactivity disorder (ADHD), autism spectrum disorder (ASD), cerebral palsy (CP), development coordination disorder (DCD), or fetal alcohol spectrum disorder (FASD), to name a few. NDD are widely defined as a “group of congenital or acquired long-term conditions that are attributed to impairment of the brain and/or neuromuscular system and create functional limitations” [2]. The impairments and dysfunctions associated with NDD are usually associated with significant consequences for children, families, and society [3,4,5,6].

Physical activity provides numerous health benefits for children [7,8]. Due to physical and psychosocial limitations, children with NDD are often less active than their typically developing peers. Less frequent participation in physical activity may manifest as missed opportunities to positively impact various health outcomes including the development of motor skills, social learning, and mental wellness [9,10,11,12,13,14,15,16]. Families of children with NDD often report the desire to identify community-based physical activity programs aimed at stimulating cognitive development and improving self-control, autonomy, social integration, and quality of life. While this type of program has been found to be associated with a range of benefits for children, variations in research design, populations studied, intervention type, and outcomes measured have made determining the true effects of this type of program difficult.

To our knowledge, no in-depth review has been conducted to explore the existing literature regarding the multiple types of community-based physical activity programs for children with varieties of NDD, and the different types of outcomes studied. A scoping review of the literature was conducted to examine the impact of physical activity on the cognitive function of children with NDD, identify the most promising interventions, and clarify existing gaps and inconsistencies in the literature. More specifically, this manuscript aimed to: (a) investigate the extent, range, and nature of research activities regarding the effect and emerging evidence in the field of physical activity interventions on cognitive development among children and youth with NDD, and (b) help identify key gaps in research and determine precise research questions for future investigations. Collectively, this information will also help to determine the value of conducting further systematic reviews and meta-analyses.

## 2. Materials and Methods

According to Colquhoun et al., “a scoping review is a form of knowledge synthesis that addresses an exploratory research question aimed at mapping key concepts, types of evidence, and gaps in research related to a defined area or field by systematically searching, selecting, and synthesizing existing knowledge” (p. 1293, [17]). Guiding documents by Tricco et al. [18] were utilized for direction. A health research librarian with expertise in literature reviews (A.R.) provided guidance at all stages of this review.

### 2.1. Search Strategy

The following electronic databases were searched: PubMed, MEDLINE (EBSCOhost), CINAHL, PsycINFO, and ERIC (Education Resources Information Center). Search terms representing physical activity or specific activities such as exercise, recreation therapy, physical activity, and sport were combined with search terms representing NDD or specific conditions such as neurodevelopmental disorders, cerebral palsy, intellectual disability, autism spectrum disorder, and fetal alcohol spectrum disorder. Controlled vocabulary was incorporated into the search strategy to increase the breadth and relevance of the articles retrieved. The searches were limited by date from 1995 to 2017. No limits were set on type of publication. Only English language articles were included. See Appendix A for a search strategy for each database.

### 2.2. Selection Criteria

We used “population” and “intervention” criteria of PICO (Population, Intervention, Comparison, Outcome) to guide the selection of articles for this scoping review. We did not use “outcome” since it was one objective of our scoping review to identify the measured outcomes. We also did not use “comparison” as we did not want to limit the scope of our review by this restriction. Articles were eligible for inclusion if they focused on children and youth aged 0–17.99 years with NDD (e.g., ADHD, ASD, CP, FASD, DCD, intellectual disabilities, etc.). We adopted a non-categorical approach that focuses on children’s needs rather than disease category. When parents decide to send their child to a physical activity program in the community, they share the same need, whatever the disease. Therefore, we did not try to identify how the diagnosis of disease was made in the different studies we analyzed in the scoping review. All types of physical activity programs were of interest (e.g., sport, exercise, physical activity, equine-assisted therapy, dance, music, leisure, etc.) as long as they were offered in the context of community-based programs. Outcomes were not pre-specified in order to identify all outcomes used in different studies. Medically based rehabilitation programs (e.g., physiotherapy or occupational therapy) were excluded because they are attached to a specific domain of practice; although effective, these interventions are limited by their domain-specific boundaries. For instance, therapeutic sessions are provided individually, which limits the child’s interactions with other children. In addition, studies with an intervention duration of less than four weeks were excluded as they were unlikely to have long lasting effects; furthermore, these short programs are often offered in the context of holiday camps and the whole special context makes it difficult to identify the effects of physical activity from the broader effects of attending the camp. Primary research studies and dissertations were included: books, letters to the editor, commentary, and protocol papers were not included. For review papers, their selected articles have been screened and relevant papers have been selected for our scoping review.

### 2.3. Screening Process and Study Selection

**Figure 1:**
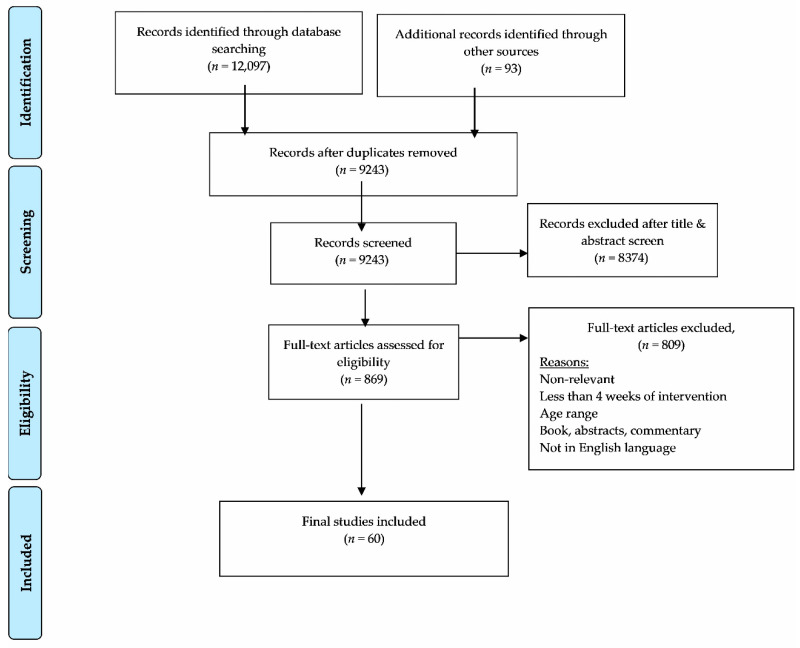
PRISMA Flow Diagram: Identification, screening, eligibility, and inclusion of studies.

Figure 1 outlines the screening process. Once the articles were identified and retrieved from the online databases and the selected articles from review papers were added, all documents were exported to RefWorks (a reference management software). Duplicates were manually removed using the referencing software. Using the pre-established selection criteria, the titles and abstracts of all retrieved articles were reviewed by two independent researchers. Retained papers were then reviewed in full by the same two independent researchers for inclusion in the review. In instances of disagreement between two reviewers (less than 1% of papers), a third reviewer was brought in as a mediator.

### 2.4. Data Extraction and Synthesis

Once the full set of included articles was ascertained, the key findings of each paper were mined and presented in a standardized extraction table. Extracted data included: general study information (author, year of publication, country, study design), study aim, population (including sample size), setting and duration, description of exposure, outcomes of interest, measurement tools, and key findings.

Data were synthesized for each study outcome as “improved”, “no change”, “regression”, or “mixed” depending on the findings of the paper. To the best of our abilities, findings were grouped based on age, gender, type of NDD, and type of physical activity exposure.

## 3. Results

### 3.1. Identified Studies

Figure 1 outlines the studies identified at each stage of the screening process. A total of 12,097 articles resulted from the initial search, with an additional 93 articles identified from the identified review papers. Upon removal of duplicates, 9243 potentially eligible articles remained. Citations were screened first by title and abstract. Of the 869 articles that made it to the full-text screening, 809 were subsequently removed as they did not meet the eligibility criteria (e.g., did not focus on the health outcome of interest, incorrect age, not published in English language, etc.). A total of 60 studies were deemed eligible for inclusion in this review.

### 3.2. Description of Included Papers

The publication dates ranged from 1987 to 2017, with most of the articles published in the United States followed by the United Kingdom and conducted mostly in the US followed by Taiwan. Table 1 summarizes the 60 studies included in this review. The mean sample size was 30 children (Standard Deviation (SD) + 25.8) and ranged from 1 to 116; about 75% had a sample size less than 43. The majority of studies (76.7%) focused on the age group of 6–12 years, followed by 13–18 years (18.3%), and 0–5 years (3.3%). Most papers (43.3%) either included male participants only, or more than 90% of their participants were male. This was followed by both male and females (51.7%), and not stated (5%). None of the studies included only females as their participants. The child’s neurodevelopmental diagnosis type varied across studies, with the vast majority focusing on ADHD (38.3%) and ASD (28.3%). Duration of physical activity interventions averaged 14 weeks (SD + 20.6) with about 75% being less than 12 weeks. Accordingly, the average study duration was 17.2 weeks (SD + 23.3) and about 75% of the studies had a duration of less than 20 weeks. There was much variability in study designs: 38.3% were randomized controlled trials (RCTs), 26.7% were quasi-experimental, followed by 13.3% prospective cohort studies, 6.7% single-subject studies, 6.7% qualitative designs, 5% case studies, 1.7% cross-sectional studies, and 1.7% retrospective cohort studies. Type of exposure also varied including physical activity and exercise (43.3%), equine-assisted therapy/hippotherapy (21.7%), yoga (11.7%), sport (11.7%) as the most frequent ones, followed by aqua therapy (5%), martial arts (3.3%), active video games (1.7%), and dance (1.7%). See Table 1 for additional details on each included study.

When the study population consisted of children with ASD, the most common intervention was equine-assisted therapy (*n* = 7) followed by exercise (*n* = 5). For children with ADHD, the most common intervention was exercise (*n* = 12) followed by yoga (*n* = 3) and equine-assisted therapy (*n* = 3).

### 3.3. Study Outcomes and Findings

Table 1 shows different cognitive function outcomes with different populations. Some papers reported on more than one outcome and the impact reported varied.

Table 2 shows the frequency of cognitive outcomes assessed and the direction of change.

Most of the reported results were measured by standardized scales. Depending on the direction of the change of scores before and after intervention, results were classified as “improvement”, “no change”, “regression”, or “mixed results”.

Attention was measured in 37 out of 60 studies (61.7%) and 30 of them (81%) reported improvement (17 were statistically significant) and 1 study had mixed results. The six studies that did not show any change in attention were mostly RCT and quasi-experimental studies of yoga or equine-assisted therapy with children diagnosed with ADHD or ASD with a sample size ranging from 7 to 24.

Cognitive control was measured in 15 studies (25%) and 13 of them (92.9%) showed positive changes (9 were statistically significant). Two studies did not show any change: one was a quasi-experimental study of equine-assisted therapy with seven boys with ASD and the other one was a quasi-experimental study of an exergame with both genders with ASD.

Working memory was the outcome reported in 8 out of 60 studies (13.3%). Seven of them reported a positive change (four were statistically significant) and one had mixed results. Cognitive flexibility has been measured in seven studies (11.7%). Six of them reported a positive change (three were statistically significant); the single study that did not report improvement was an RCT of exercise intervention with 44 boys and girls with Down syndrome. Eighty per cent and 75% studies that measured language and academic achievement, respectively, reported favorable effects of physical activity interventions.

Other cognitive function outcomes included cognitive performance, cognitive process, academic engagement and performance, planning, fluid intelligence, alertness, and speed; all studies that assessed these functions noted positive changes. We did not find any research study that investigated the effect of physical activity on memory (short- and long-term) and perceptual processing. Different outcomes were selected for different NDD conditions. Table 3 shows the frequency of cognitive outcomes assessed in children with ADHD, ASD, CP, and Down syndrome and the direction of change.

Specific to ADHD, attention was measured in 19 studies and 16 (84.2%) reported positive changes. Working memory and cognitive control were studied in four studies that all showed improvement. In studies of children diagnosed with ASD, 9 studies out of 37 (24.3%) measured attention, with 6 of them showing positive changes. Cognitive control was assessed in four studies, with two studies reporting favorable effects.

Additionally, different interventions were associated with specific outcomes. For instance, the effects of exercise (*n* = 26 studies) were assessed on attention in 11 articles and all reported positive changes. In addition, working memory has been measured in seven articles, with six reporting improvement. For equine-assisted therapy interventions (*n* = 13 studies), attention was measured in 9, with 5 reporting improvement. For language (*n* = 3) and cognitive control (*n* = 3), two studies for each of these outcomes showed positive changes (see Table 4).

## 4. Discussion

This comprehensive scoping review reports the impact of physical activity on the cognitive functions of children and youth with different types of NDD conditions: ASD, ADHD, CP, Down syndrome, intellectual disabilities, physical disabilities, behavioral and social disabilities, learning disabilities, and developmental coordination disorder (DCD). A number of cognitive function outcomes were explored: executive function including cognitive flexibility, cognitive control and working memory, attention, short- and long-term memory, learning, perceptual processing, and alertness.

Most studies reported improvement in their measured cognitive function outcomes. About half of the reported improvements were statistically significant. No studies reported negative impacts or symptom regression on cognition among child participants with NDD. The majority of studies used standardized measurement tools in order to assess the change in studied outcomes. This finding indicates the role of physical activity in the learning and development of a child with NDD. The major findings of this scoping review will be discussed in the following paragraphs.

Most of the included studies have been conducted with boys 6–12 years of age who were diagnosed with ADHD (*n* = 23, 38.3%). This finding certainly corresponds to the fact that the main challenges described are triggered in the context of school exposure, and ADHD incidence is much higher among boys than girls. This focus of interest indicates the importance of identifying effective interventions for school age children with NDD. Regarding gender, most studies focus on boys, which corresponds to the fact that boys are more often diagnosed with ADHD and ASD in comparison to girls; however, we cannot eliminate a possible gender bias with more boys willing to participate in these studies. While some studies reported small improvements in girls with NDD, additional work is needed to clarify the relationship between physical activity and this particular sub-population.

Among the 60 studies that were included in the present scoping review, the largest number of positive impacts of physical activity was found in studies of exercise interventions followed by equine-assisted therapy, then sports. Of these papers, the majority focused on children with ADHD and ASD, where attention and its sub-domains of focus and concentration were the most commonly measured cognitive function. This finding corresponds to the main concern in this population, especially in the school context [76,77,78]. In the case of ADHD, participation in high-intensity physical activity or exercise may increase the release of endorphins (which helps regulate mood, pleasure, and pain) and neurotransmitters like dopamine, norepinephrine, and serotonin levels (which positively affect focus and attention) [79]. Combined, these effects on the brain help increase alertness and reduce the craving for new stimuli, which are typical characteristics of children with ADHD. Similarly, with ASD, higher-intensity physical activity has been noted in the literature as an effective supplement to children’s treatment regimens [80,81,82]. Studies show that moderate-to-vigorous physical activity is associated with decreases in self-stimulatory behaviors, hyperactivity, aggression, self-injury, and destructiveness [83]. Furthermore, as many children with ASD are at increased risk for weight gain, including regular physical activity in their daily routine may have beneficial effects [84,85]. The incorporation of animal therapy into the treatment of protocols of children with ADHD and ASD is well-received and its positive effects have been noted in other reviews [86,87].

The type of NDD diagnosis and physical activity intervention were examined in this review. We found that children with ASD saw the most improvements in attention and that equine-therapy appeared to be the best type of activity to produce such changes. As for children with CP, most gains were reported in the attention domain, with aqua therapy and movement therapy serving as ideal activities to realize such improvements. Lastly, children with ADHD reported improvements in attention, with again, exercise and movement therapy serving as the preferred conduits to improved cognitive functions, memory, and development. The acquisition of such information is paramount to our understanding of which domains of cognitive function are most positively impacted by physical activity and by which types of activity exposures. Consequently, this information will assist with the creation of tailored physical activity programming for children with NDD based on their unique abilities.

Our findings confirm the ones from other literature reviews. Pontifex et al. [88] in their narrative review examined the role of physical activity in reducing barriers to learning in children with developmental disorders including ADHD and ASD. Findings indicated that both single bouts of activity and chronic physical activity were associated with improved classroom performance [88,89]. A meta-analysis of 22 articles by Tan et al. [89] found an overall small to medium effect of exercise on cognition. Their findings support the efficacy of exercise interventions in enhancing certain aspects of cognitive performance in individuals with ASD and/or ADHD. In another systematic review and meta-analysis of eight RCTs, Cerrilo-Urbina et al. [90] reported that short-term aerobic exercise, based on several aerobic intervention formats, seems to be effective for mitigating symptoms such as attention, hyperactivity, impulsivity, anxiety, executive function, and social disorders in children with ADHD. However, to our knowledge, this scoping review is the first comprehensive review that explored the impacts of several types of physical activity interventions on all aspects of cognitive function for children with a variety of neurodevelopmental challenges.

### 4.1. Strengths and Limitations

The majority of papers included in this review were RCTs and quasi-experimental studies, which highlights the credibility of the overall evidence. In addition, having a research question that was broad in scope allowed us to investigate many aspects of the existing relevant research studies. Despite these strengths, there are several limitations of this review worth noting. First, most of the studies had small sample sizes (75% of studies included less than 43 participants) and the physical activity programs were short in duration (75% of studies had interventions less than 12 weeks). Second, most studies did not organize data collection by a person who was unaware of the intervention status, which makes the studies prone to different types of observation/report biases and Hawthorne effects (i.e., the change in behavior of study participants due to the awareness of being observed) [91,92]. Third, information was generally lacking regarding the child/family’s satisfaction of the effect and the level that the positive impacts of physical activity could meet the specific needs of a child and family. Fourth, due to the vast variability in reporting interventions and results, informing best practice recommendations is not possible. Finally, although the current review considered solely English-language, peer-reviewed publications and academic gray literature, examining the international and non-academic gray literature may help to expand and deepen our understanding of physical activity on acquisition of new functions (cognitive and psychological) and learning among children with NDD.

### 4.2. Future Directions

Based on the findings of this scoping review, we have identified several areas for further investigations. High quality studies on the impact of physical activity on brain function among very young children (under 6 years) with NDD are appealing. Brain plasticity is maximal in young children [93,94]; therefore, interventions should target younger children instead of waiting for impairments to be revealed in light of the demands of the school classroom. More generally, specific research questions include determining the type of activities at different ages and which effect is expected on different types of outcomes. A more focused look at the relationship between physical activity and brain function across disability categories including the undertaking of further investigations into the development of physical literacy for long-term physical activity and its impact on brain health would also be of interest. Given that peer relationships become increasingly important from childhood to adolescence, additional research to explore the impact of physical activity on social inclusion and personal identity development for children with NDD is important. As well, investigating the level of child/family’s satisfaction of the positive impacts is of paramount importance. Lastly, a rigorous systematic review and meta-analysis of RCTs that investigate the effects of physical activity on children and youth with NDD is warranted.

## 5. Conclusions

The findings of this scoping review highlight that physical activity may have a favorable impact on the cognitive outcomes of children and youth with NDD. Given these noted benefits, additional investigations are needed to help optimize the use of physical activity in the daily lives of children with NDD to not only support improved cognitive functions, but overall social integration and quality of life as well.

## Figures and Tables

**Table 1:** Summary of study characteristics.

Study	Design	Sample Size	Age Group *	Gender **	PD	Activity Type and Setting	SD	Diagnosis	Results (Positive [+], Negative/no change [−], Mixed [−/+])
Ahmed et al. (2011) [19]	RCT	84	13–18	Male and Female	10	Exercise (Included upper limb, lower limb, trunk, and neck aerobic exercises in addition to free running) Setting: School and home	10	ADHD	(+) Attention
Alesi et al. (2014) [20]	Single subject study design	3	13–18	Male and Female	8	Exercise (Integrated group exercise training with goal setting) Setting: Not mentioned	24	Down syndrome	(−/+) Working memory (+) Alertness and speed
Bass et al. (2009) [16]	Quasi-experimental	34	6–12	Male and Female	12	Equine-assisted therapy Setting: Good Hope Equestrian Training Centre	12	ASD	(+) Attention
Borgi et al. (2016) [21]	RCT	28	6–12	Male	24	Equine-assisted therapy Setting: Equine-assisted therapy center	24	ASD	(+) Planning
Bowling et al. (2017) [22]	RCT	103	6–12	Male and Female	7	Exercise (Cybercycling during physical education classes) Setting: School	24	Behavior disorders	(+) Self-regulation
Bustamante et al. (2016) [23]	RCT	35	6–12	Male and Female	10	Exercise (Physically active games and modified sports) Setting: School	13	Disruptive Behavior disorders and ADHD	(+) Cognitive control (+) Working memory (+) Cognitive flexibility
Chambers et al. (2016) [24]	Single subject Study design	5	6–12	Male and Female	4	Exercise (short-burst high-intensity physical exercise such as lunges, side jumps, and Chinese push-ups) Setting: School	4	ADHD	(+) Working memory (+) Attention
Chang et al. (2014) [25]	Quasi-experimental	27	6–12	Male and Female	8	Aqua therapy Setting: Local swimming pool	Not Stated	ADHD	(+) Response inhibition
Choi et al. (2015) [26]	RCT	50	13–18	Male	6	Exercise (Aerobic exercises consisted of running (shuttle run, zigzag run), jumping rope (individual and group jumps), and basketball (dribble, pass, shoot, and game) Setting: Not mentioned	6	ADHD	(+) Cognitive flexibility (+) Attention
Chou et al. (2017) [27]	Quasi-experimental	49	6–12	Male and Female	8	Yoga Setting: Dance studio	8	ADHD	(+) Attention
Cleary et al. (2017) [28]	Qualitative	28	Students: 13–18	Students: Male and Female	9	Exercise (Included indoor activities (such as treadmill, stationary bicycle, stepping machine) and outdoor activities (such as running, sprinting drills, or cycling in the school grounds) Setting: School	8	Students’ diagnosis: Cerebral palsy	(+) Attention
Cuypers et al. (2011) [29]	Quasi-experimental	5	6–12	Male	8	Equine-assisted therapy Setting: Riding School	24	ADHD	(+) Attention
Gabriels et al. (2015) [30]	RCT	116	6–12	Male and Female	10	Equine-assisted therapy Setting: Riding Centre	14	ASD	(+) Self-regulation
Gabriels et al. (2012) [15]	Quasi-experimental	42	6–12	Male and Female	10	Equine-assisted therapy Setting: Colorado Therapeutic Riding Centre	18	ASD	(+) Self-regulation
Garcia-Gomez et al. (2016) [31]	Quasi-experimental	14	6–12	Male and Female	13	Equine-assisted therapy Setting: Monfragüe Equestrian Centre of Cáceres	Not Stated	ADHD	(−) Attention
Garcia-Gómez et al. (2014) [12]	Quasi-experimental	16	6–12	Male and Female	12	Equine-assisted therapy Setting: Monfragüe Equestrian Centre of Cácere	12	ASD	(−) Attention
Garg et al. (2013) [32]	Prospective cohort study	51	6–12	Male and Female	26	Yoga Setting: Classroom	26	Multiple handicapping conditions, developmental disabilities, or ASD	(+) Self-regulation (+) Attention
Grönlund et al. (2005) [33]	Quasi-experimental	2	6–12	Male	12	Dance Setting: Clinic	12	ADHD	(−/+) Attention
Hariprasad et al. (2013) [34]	Prospective Cohort	9	6–12	Male	8	Yoga Setting: Child Psychiatry Unit	12	ADHD	(+) Attention
Hartshorn et al. (2001) [35]	Quasi-experimental	38	0–5	Not Stated	8	Exercise (Involved using hoops and jumping in and out of them, putting different body parts in and out of the hoops, following the therapist through an obstacle course of different shape and different height gym mats, and moving to a tambourine and stopping when the tambourine stopped) Setting: Not mentioned	8	ASD	(+) Attention
Hilton et al. (2014) [36]	Quasi-experimental	7	6–12	Male and Female	10	Exergame Setting: Clayton Child Centre	10	ASD	(+) Working memory (+) Alertness and speed
Hulls et al. (2006) [37]	Cross-sectional	18 occupational therapists	Children: 6–12	Children: Not Stated		Activity Type: Aqua therapy Setting: Not mentioned	Not Stated	ASD	(+) Attention
Jang et al. (2015) [38]	Quasi-experimental	20	6–12	Male	12	Equine-assisted therapy Setting: Equine Facility	14	ADHD	(+) Attention
Jenkin et al. (2013) [39]	Quasi-experimental	7	6–12	Male	8	Equine-assisted therapy Setting: Reserved are at a riding facility	9	ASD	(−) Cognitive control(−) Attention(−) Language
Jensen et al. (2004) [40]	RCT	19	6–12	Male	20	Yoga Setting: Westmead hospital	20	ADHD	(−) Attention
Johansson et al.(1998) [41]	Retrospective Cohort	92	13–18	Male	156	Exercise (Physical education) Setting: Pacific Northwest program	156	Social and emotional problems	(+) Academic achievement
Kaiser et al. (2006) [42]	Prospective Cohort	31	13–18	Male and Female	6	Equine-assisted therapy Setting: CHUM Riding Therapeutic Centre	6	Cerebral palsy, emotional impairment, learning disability	(+) Attention
Kang et al. (2011) [43]	RCT	28	6–12	Male	6	Sport (Sport therapy program) Setting: Department of Psychiatry of Chung Ang University Medical Centre	6	ASD	(+) Attention
Lawson et al. (2012) [44]	Single subject study design	3	0–5	Male	5	Yoga Setting: The Preschool and Language Stimulation Program School	7	Speech and developmental delay, behavioral problem, ADHD	(+) Attention
Lufi et al. (2011) [45]	Quasi-experimental	32	6–12	Male	52	Sport (Sport-based group therapy such as basketball and running) Setting: Not mentioned	104	Behavioral disorders	(+) Attention
Macauley et al. (2004) [46]	Prospective cohort	3	6–12	Male	6	Hippotherapy Setting: Merlin Farms Equestrian Center	6	Learning disability	(+) Language
Mackinnon et al. (1995) [47]	RCT	19	6–12	Male and Female	26	Equine-assisted therapy Setting: Therapeutic riding program	26	Cerebral palsy	(−) Attention
MacMahon et al. (1987) [48]	RCT	54	6–12	Male	20	The aerobic group’s exercise consisted of distance running, aerobic dance, and variants of soccer Setting: Not mentioned	20	Learning disability	(−) Academic achievement
Majorek et al. (2004) [49]	Case series	5	6–12	Male	36	Exercise (Therapeutic Eurythmy-movement therapy) Setting: Therapy rooms of a pediatrician’s surgery	36	ADHD	(+) Attention
McKune et al. (2003) [50]	Quasi-experimental	19	6–12	Male and Female	5	Exercise (Included activities such as relay runs, simple plyometric exercises, running through a mini obstacle course, a distance run of 1–2 km, and skipping) Setting: Home	9	ADHD	(+) Attention
Memarmoghaddam et al. (2016) [51]	RCT	40	6–12	Male	8	Exercise (Aerobic and goal directed exercises) Setting: University Sports Hall	8	ADHD	(+) Cognitive inhibition
Milligan et al. (2015) [52]	Qualitative	46	Youth: 13–18	Youth: Male and Female	20	Martial Arts Setting: Child Development Institute	20	Learning disability	(+) Self-regulation
Morand et al. (2004) [53]	RCT	18	6–12	Male	12	Martial Arts Setting: Martial arts school	12	ADHD	(+) Academic performance (+) Attention
Neely et al. (2015) [54]	Case study	2	6–12	Male and Female	12	Exercise (Jumping on an indoor trampoline) Setting: Classroom	12	ASD	(+) Academic engagement
Nicholson et al.(2011) [55]	Single subject study design	4	6–12	Male	6	Exercise (Jogging) Setting: Classroom	6	ASD	(+) Academic engagement
O’Callaghan et al. (2003) [56]	RCT	4	6–12	Male and Female	6	Sport (Kickball) Setting: Campus of a large public university	6	ADHD	(+) Attention
Ozer et al. (2012) [57]	RCT	76	13–18	Male	8	Sport (Soccer) Setting: Soccer Field	8	Intellectual disability	(+) Attention
Ozer et al. (2007) [58]	RCT	23	6–12	Male and Female	14	Aqua therapy Setting: Swimming pool	38	Cerebral palsy	(+) Attention
Packard et al. (2007) [59]	Prospective Cohort	10	6–12	Male and Female	4	Exercise (Vigorous aerobic physical activity) Setting: School	6	ADHD	(+) Attention
Pan et al. (2017) [60]	RCT	22	6–12	Male	12	Exercise (Motor and movement skills related to table tennis) Setting: Gymnasium	24	ASD	(+) Cognitive flexibility
Pan et al. (2016) [61]	RCT	32	6–12	Male	12	Sport (Racket sport) Setting: Table tennis center	24	ADHD	(+) Cognitive flexibility (+) Attention (+) Cognitive process
Porter et al. (2013) [62]	Case study	1	6–12	Male	4	Yoga Setting: Classroom	4	ASD	(+) Attention
Ringenbach et al. (2016) [63]	RCT	44	13–18	Male and Female	8	Exercise (Assisted cycling therapy) Setting: Cycling Bike	8	Down syndrome	(+) Response inhibition (−) Cognitive flexibility (+) Language (+) Alertness and speed
Rosenblatt et al. (2011) [64]	Prospective Cohort	24	6–12	Male	8	Yoga Setting: Medical school teaching hospital	8	ASD	(−) Attention
Rosenthal-Malek et al. (1997) [65]	Quasi-experimental	5	13–18	Male	10	Exercise (Mildly strenuous jogging) Setting: Urban public school and a community-based workshop	10	ASD	(+) Academic performance
Smith et al. (2013) [66]	Prospective Cohort	17	6–12	Male and Female	9	Exercise (Continuous moderate-to-vigorous physical activity such as hopping, skipping, etc.) Setting: Not mentioned	11	ADHD	(+) Response inhibition (+) Working memory
Stickney et al. (2010) [67]	Qualitative	34	Children: Not Stated	Children: Not stated	8	Equine-assisted therapy Setting: Central Kentucky Riding for Hope	8	ASD	(+) Attention (+) Language (+) Academic achievement
Tsai et al. (2009) [68]	RCT	43	6–12	Male and Female	10	Sport (Tennis) Setting: A laboratory	10	Developmental coordination disorder	(+) Inhibitory control
Tsai et al. (2012) [69]	RCT	51	6–12	Male and Female	10	Sport (Soccer) Setting: School	10	Developmental coordination disorder	(+) Inhibitory control (+) Attention
Tsai et al. (2014) [70]	RCT	60	6–12	Male and Female	16	Exercise (The endurance training program consisted of interval training and one continuous long-distance running session, and one session with another aerobic activity (e.g., cycling, step aerobics, or rope jumping)) Setting: School	16	Developmental coordination disorder	(+) Working memory
Verret et al. (2012) [71]	Quasi-experimental	21	6–12	Male	10	Exercise (Sessions included warm-up; progressive aerobic, muscular, and motor skills exercises; and cool down) Setting: School gymnasium	12	ADHD	(+) Attention
Wehrle et al. (2017) [72]	Qualitative	6	6–12	Male	8	Exercise (Includes warm-up, free play, and skill of a week) Setting: Shady Lane Elementary School	8	ADHD	(+) Attention (+) Academic performance
Wendt et al. (2000) [73]	Quasi-experimental	24	6–12	Male and Female	6	Exercise (Intense Aerobic-type physical activity program) Setting: SUNY Buffalo Amherst, Turf Football Stadium	6	ADHD	(+) Cognitive control (+) Working memory (+) Cognitive flexibility (+) Attention (+) Cognitive performance (+) Language (+) Cognitive process (+) Planning (+) Academic achievement (+) Fluid intelligence
Yildirim et al. (2010) [74]	RCT	50	13–18	Male and Female	12	Exercise (Circuit training, resistance training, and interval speed training) Setting: School	12	Intellectual disability	(+) Alertness and speed
Ziereis et al. (2015) [75]	RCT	43	6–12	Male and Female	12	Exercise (Various activities such as juggling, tennis, trampoline, juggling, etc.) Setting: Facilities of the University of Regensburg’s Institute for Sport Science	14	ADHD	(+) Working memory

Notes: PD = Program Duration in weeks; SD = Study Duration in weeks; RCT = Randomized Controlled Trials; ADHD = Attention-Deficit/Hyperactivity Disorder; ASD = Autism Spectrum Disorder. * We categorized the age to three groups including “0–5”, “6–12”, and “13–18”-year-olds. Each study has been assigned the age group based on the mean age of study participants; ** Study population has been considered as “Male” when all or more than 90% of study participants were male. Similarly, when all or more than 90% of study participants were female, the study population has been considered as “Female”.

**Table 2:** Frequency of cognitive outcomes assessed and the direction of change.

Outcomes	Frequency *n* (%)			
	Included Studies *	Improvement **	No Change **	Mixed **
Cognitive flexibility	7 (11.7)	6 (85.7)	1 (14.3)	
Cognitive control	15 (25)	13 (86.7)	2 (13.3)	
Working Memory	8 (13.3)	7 (87.5)	0	1 (12.5)
Attention	37 (61.7)	30 (81.1)	6 (16.2)	1 (2.7)
Language	5 (8.3)	4 (80)	1 (20)	
Cognitive performance	1 (1.7)	1 (100)	0	
Cognitive process	2 (3.3)	2 (100)	0	
Planning	3 (5)	3 (100)	0	
Academic Achievement	4 (6.7)	3 (75)	1 (25)	
Academic Engagement	2 (3.3)	2 (100)	0	
Academic Performance	3 (5)	3 (100)	0	
Fluid intelligence	1 (1.7)	1 (100)	0	
Alertness and Speed	4 (6.7)	4 (100)	0	

* The percentage indicates the proportion of included studies that assessed each of the listed cognitive outcomes; ** The percentage indicates the proportion of included studies that showed improvement, no change, or mixed results when the study assessed the relevant outcome.

**Table 3:** Frequency of cognitive outcomes assessed in children with attention-deficit/hyperactivity disorder (ADHD), autism spectrum disorder (ASD), cerebral palsy (CP), and Down syndrome, and the direction of change.

	ADHD		ASD		CP		Down Syndrome	
Outcomes (Total Number of Studies)	Frequency *n* (%)	Number of Positive Results	Frequency *n* (%)	Number of Positive Results	Frequency *n* (%)	Number of Positive Results	Frequency *n* (%)	Number of Positive Results
Cognitive flexibility (7 studies)	3 (42.8)	3	2 (28.6)	2	0 (0)	0	1(14.3)	0
Cognitive control (15 studies)	4 (26.7)	4	4 (26.7)	2	0 (0)	0	1(6.7)	1
Working Memory (8 studies)	4 (50)	4	1 (12.5)	1	0 (0)	0	1(12.5)	0
Attention (37 studies)	19 (51.4)	16	9 (24.3)	6	3 (8.1)	2	0 (0)	0
Language (5 studies)	1 (20)	1	2 (40)	1	0 (0)	0	1(20)	1
Cognitive performance (1 study)	1 (100)	1	0 (0)	0	0 (0)	0	0 (0)	0
Cognitive process (2 studies)	2 (100)	2	0 (0)	0	0 (0)	0	0 (0)	0
Planning (3 studies)	1 (33.3)	1	2 (66.7)	2	0 (0)	0	0 (0)	0
Academic Achievement (4 studies)	1 (25)	1	1(25)	1	0 (0)	0	0 (0)	0
Academic Engagement (2 studies)	0 (0)	0	2 (100)	2	0 (0)	0	0 (0)	0
Academic Performance (3 studies)	2 (66.6)	2	1 (33.3)	1	0 (0)	0	0 (0)	0
Fluid intelligence (1 study)	1 (100)	1	0 (0)	0	0 (0)	0	0 (0)	0
Alertness and Speed (4 studies)	0 (0)	0	1 (25)	1	0 (0)	0	2 (50)	2

**Table 4:** Frequency of cognitive outcomes assessed for the two most common physical activity interventions and the direction of change.

	Physical Activity/Exercise/Movement Therapy		Equine-Assisted Therapy	
Outcomes (Total Number of Studies)	Frequency *n* (%)	Number of Positive Results	Frequency *n* (%)	Number of Positive Results
Cognitive flexibility (7 studies)	5 (71.4)	4	0 (0)	0
Cognitive control (15 studies)	6 (40)	6	3 (20)	2
Working Memory (8 studies)	7 (87.5)	6	0 (0)	0
Attention (37 studies)	11 (29.7)	11	9 (24.3)	5
Language (5 studies)	2 (40)	2	3 (60)	2
Cognitive performance (1 study)	1 (100)	1	0 (0)	0
Cognitive process (2 studies)	1 (50)	1	0 (0)	0
Planning (3 studies)	1 (33.3)	1	1 (33.3)	1
Academic Achievement (4 studies)	3 (75)	2	1 (25)	1
Academic Engagement (2 studies)	2 (100)	2	0 (0)	0
Academic Performance (3 studies)	2 (66.7)	2	0 (0)	0
Fluid intelligence (1 study)	1 (100)	1	0 (0)	0
Alertness and Speed (4 studies)	3 (75)	3	1(25)	1

## Appendix A. Search Strategy for Each Individual Database

*Pub Med*

“Recreation Therapy” [Mesh] OR “Music Therapy” [Mesh] OR “Play Therapy”[Mesh] OR “Leisure Activities”[Mesh] OR “Recreation”[Mesh] OR “Art Therapy”[Mesh] OR “Exercise Therapy”[Mesh] OR “Sports”[Mesh] OR “Animal Assisted Therapy”[Mesh] OR “Equine-Assisted Therapy”[Mesh] OR “Gymnastics”[Mesh] OR “Dance Therapy”[Mesh] OR “aquatic therapy” [tiab] OR “hydro therapy” [tiab] OR “creative arts therapy” [tiab] OR “Exercise”[Mesh] OR “therapeutic recreation” [tiab] OR “recreation” [tiab] OR “exercise” [tiab] OR “exercises” [tiab] OR “physical activities” [tiab] OR “physical activity” [tiab] OR “sport” [tiab] OR “sports” [tiab].

AND

“Intellectual Disability” [Mesh] OR “Disabled Persons” [Mesh] OR “Developmental Disabilities” [Mesh] OR “Rett Syndrome” [Mesh] OR “Down Syndrome” [Mesh] OR “Autistic Disorder” [Mesh] OR “Autism Spectrum Disorder” [Mesh] OR “Cerebral Palsy” [Mesh] OR “Attention Deficit Disorder with Hyperactivity” [Mesh] OR “Fetal Alcohol Spectrum Disorders”[Mesh] OR “neurodevelopment disorder” [tiab] OR “neurodevelopment delay” [tiab] OR “neurodisability” [tiab] OR “Neurodevelopmental Disorders”[Mesh] OR “disability” OR “neurodevelopmental disabilities” OR “neurodevelopmental delays” OR “neurodevelopmental disorders” OR “neurodevelopmental disability” OR “neurodevelopmental disorder” OR “disabilities” OR “neurodevelopmental delay” OR “neurodisabilities”.

Limitations: 1995–2017, All child (birth–18), English.

*MedLine Search History*

(MH “Recreation Therapy”) OR (MH “Leisure Activities”) OR (MH “Recreation”) OR (MH “Hydrotherapy”) OR (MH “Art Therapy”) OR “Creative Arts Therapy” OR “Therapeutic Exercise” OR (MH “Pet Therapy”) OR (MH “Equine Assisted Therapy”) OR (MH “Dance Therapy”) OR “Drama Therapy” OR (MH “Play Therapy”) OR (MH “Music Therapy”) OR (MH “Gymnastics”) OR (MH “Yoga”) OR (MH “Martial Arts”) OR (MH “Skating”) OR (MH “Skiing”) OR (MH “Baseball”) OR (MH “Basketball”) OR (MH “Soccer”) OR (MH “Recreation”) OR (MH “Dancing”) OR (MH “Racquet Sports”) OR (MH “Snow Sports”) OR (MH “Sports for Persons with Disabilities”) OR (MH “Sports”) OR (MH “Exercise+”) OR (MH “Movement”) OR (MH “Motor Activity”) OR TI “therapeutic recreation” OR AB “therapeutic recreation” OR TI recreation OR AB recreation OR TI exercise OR AB exercise OR TI “physical activities” OR AB “physical activities” OR TI sport OR AB sport OR TI “physical activity” OR AB “physical activity”.

AND

(MH “Developmental Disabilities”) OR (MH “Autistic Disorder”) OR (MH “Child Development Disorders, Pervasive”) OR “Neurodevelopmental Disability” OR “Neurodevelopmental Delay” OR (MH “Rett Syndrome”) OR (MH “Cerebral Palsy”) OR (MH “Down Syndrome”) OR (MH “Intellectual Disability”) OR (MH “Attention Deficit Disorder”) OR (MH “Attention Deficit Disorder with Hyperactivity”) OR (MH “Fetal Alcohol Spectrum Disorders”) OR (MH “Cerebral Palsy”) OR (MH “Neurodevelopmental Disorders”) OR (MH “Attention Deficit and Disruptive Behavior Disorders”) OR (MH “Motor Skills Disorders”) OR disability OR “Neurodevelopmental Disabilities” OR “Neurodevelopmental Delays” OR Neurodisability OR “Neurodevelopmental Disorders” OR “Neurodevelopmental Disorder” OR (MH “Disabled Children”).

Limitations: 1995–2017, All child (birth–18), English.

*CINAHL Search History (CINAHL—Cumulative Index to Nursing and Allied Health Literature)*

(MH “Recreation Therapy”) OR (MH “Leisure Activities”) OR (MH “Recreation”) OR (MH “Hydrotherapy”) OR (MH “Art Therapy”) OR “Creative Arts Therapy” OR (MH “Therapeutic Exercise”) OR (MH “Pet Therapy”) OR (MH “Horseback Riding”) OR “Equine Assisted Therapy” OR (MH “Dance Therapy”) OR (MH “Play Therapy”) OR (MH “Music Therapy”) OR (MH “Gymnastics”) OR (MH “Yoga”) OR (MH “Martial Arts”) OR (MH “Skating”) OR (MH “Skiing”) OR (MH “Baseball”) OR (MH “Basketball”) OR (MH “Soccer”) OR (MH “Walking”) OR (MH “Dancing”) OR (MH “Recreational Therapy”) OR (MH “Aquatic Sports”) OR (MH “Extreme Sports”) OR (MH “Animal Sports”) OR (MH “Winter Sports”) OR (MH “Wheelchair Sports”) OR (MH “Race Walking”) OR (MH “Racquet Sports”) OR (MH “Rock Climbing”) OR (MH “Motor Sports”) OR (MH “Running”) OR (MH “Sports, Disabled”) OR (MH “Team Sports”) OR (MH “Track and Field”) OR (MH “Weight Lifting”) OR (MH “Bowling”) OR (MH “Endurance Sports”) OR (MH “Cycling”) OR (MH “Sports”) OR (MH “Golf”) OR (MH “Handball”) OR (MH “Exercise”) OR (MH “Physical Activity”) OR TI “therapeutic recreation” OR AB “therapeutic recreation” OR TI recreation OR AB recreation OR TI exercise OR AB exercise OR TI “physical activities” OR AB “physical activities” OR TI sport OR AB sport OR TI “physical activity” OR AB “physical activity”.

AND

(MH “Intellectual Disability”) OR (MH “Developmental Disabilities”) OR (MH “Child, Disabled”) OR “Developmental Delay” OR (MH “Autistic Disorder”) OR (MH “Child Development Disorders, Pervasive”) OR “Neurodevelopmental Disorder” OR (MH “Rett Syndrome”) OR “Neurodisability” OR (MH “Cerebral Palsy”) OR (MH “Down Syndrome”) OR “Attention Deficit Disorder” OR (MH “Attention Deficit Hyperactivity Disorder”) OR (MH “Fetal Alcohol Disorder”) OR disability OR “Neurodevelopmental Disabilities” OR “Neurodevelopmental Delays” OR Neurodisability OR “Neurodevelopmental Disorders” OR “Neurodevelopmental Disability” OR (MH “Learning Disorders”) OR (MH “Motor Skills Disorders”) OR (MH “Child Development Disorders”).

Limitations: 1995–2017, Age Groups: Infant, Newborn: birth–1 month; Infant: 1–23 months; Child, Preschool: 2–5 years; Child: 6–12 years; Adolescent: 13–18 years; English.

*PsychInfo Search History*

DE “Recreation Therapy” OR DE “Leisure Time” DE “Recreation” OR DE “Art Therapy” OR DE “Creative Arts Therapy” OR DE “Exercise” OR DE “Sports” OR DE “Animal Assisted Therapy” OR DE “Dance Therapy” OR DE “Play Therapy” OR DE “Music Therapy” OR “equine assisted therapy”“gymnastics” OR “martial arts” OR “skating” OR “skiing” OR “hockey” OR “baseball” OR “soccer” OR “yoga” OR “basketball” OR DE “Physical Activity” OR DE “Aerobic Exercise” OR DE “Weightlifting” OR DE “Yoga” OR DE “Movement Therapy” OR DE “Baseball” OR DE “Basketball” OR DE “Extreme Sports” OR DE “Football” OR DE “Judo” OR DE “Martial Arts” OR DE “Soccer” OR DE “Swimming” OR DE “Tennis” OR DE “Weightlifting” OR DE “Childrens Recreational Games” OR TI “therapeutic recreation” OR AB “therapeutic recreation” OR TI recreation OR AB recreation OR TI exercise OR AB exercise OR TI “physical activities” OR AB “physical activities” OR TI sport OR AB sport OR TI “physical activity” OR AB “physical activity”.

AND

DE “Developmental Disabilities” OR DE “Autism Spectrum Disorders” OR “Neurodevelopmental Disability” OR “Neurodevelopmental Delay” OR DE “Rett Syndrome” OR DE “Cerebral Palsy” OR DE “Down’s Syndrome” OR DE “Intellectual Development Disorder” OR DE “Attention Deficit Disorder” OR DE “Attention Deficit Disorder with Hyperactivity” OR DE “Fetal Alcohol Syndrome” OR DE “Disabilities” OR DE “Learning Disabilities” OR DE “Multiple Disabilities” OR disability OR “Neurodevelopmental Disabilities” OR “Neurodevelopmental Delays” OR Neurodisability OR “Neurodevelopmental Disorder” OR “Neurodevelopmental Disorders”.

Limitations: 1995–2017, Age Groups: Childhood (birth–12 yrs), Adolescence (13–17 yrs), English.

*ERIC Search History*

DE “Therapeutic Recreation” OR DE “Play Therapy” OR DE “Leisure Time” OR DE “Recreation” OR “Aquatic Therapy” OR DE “Art Therapy” OR “Creative Arts Therapy” OR DE “Exercise” OR “gymnastics” OR “martial arts” OR “skating” OR “skiing” OR “hockey” OR “baseball” OR “soccer” OR “yoga” OR “basketball” OR “sport” OR “Animal Assisted Therapy” OR “Pet Therapy” OR “Equine Assisted Therapy” OR “Dance Therapy” OR DE “Music Therapy” OR DE “Physical Activities” OR DE “Athletics” OR DE “Physical Recreation Programs” OR DE “Playground Activities” OR DE “Recreational Activities” OR DE “Racquet Sports” OR DE “Aquatic Sports” OR DE “Team Sports” OR TI “physical activity” OR AB “physical activity” OR TI “therapeutic recreation” OR AB “therapeutic recreation” OR TI “recreation” OR AB “recreation” OR TI “exercise” OR AB “exercise” OR TI “physical activities” OR AB “physical activities”.

AND

“Intellectual Disability” OR DE “Mental Retardation” OR “Neurodevelopmental Delay” OR “Neurodevelopmental Disability” OR DE “Autism” OR DE “Pervasive Developmental Disorder” OR DE “Attention Deficit Disorder” OR “Attention Deficit Hyperactivity Disorder” OR DE “Fetal Alcohol Syndrome” OR “Rett Syndrome” OR DE “Down Syndrome” OR DE “Disabilities” OR DE “Adventitious Impairments” OR DE “Behavior Disorders” OR DE “Communication Disorders” OR DE “Congenital Impairments” OR DE “Developmental Disabilities” OR DE “Hearing Impairments” OR DE “Injuries” OR DE “Intellectual Disability” OR DE “Language Impairments” OR DE “Learning Disabilities” OR DE “Mental Disorders” OR DE “Multiple Disabilities” OR DE “Perceptual Impairments” OR DE “Physical Disabilities” OR DE “Special Health Problems” OR DE “Speech Impairments” OR DE “Visual Impairments” OR “cerebral palsy” OR “disability” OR “disabilities”.

AND

DE “Children” OR DE “Pediatrics” OR “Adolescents” OR “Youth”

Limitations: 1995–2017, English.
